# Dihydrolycorine Attenuates Cardiac Fibrosis and Dysfunction by Downregulating Runx1 following Myocardial Infarction

**DOI:** 10.1155/2021/8528239

**Published:** 2021-10-23

**Authors:** Tingjuan Ni, Xingxiao Huang, Sunlei Pan, Zhongqiu Lu

**Affiliations:** ^1^Department of Emergency Intensive Care Unit, The First Affiliated Hospital, Wenzhou Medical University, Wenzhou, Zhejiang, China; ^2^Department of Cardiology, Zhejiang University, Hangzhou, Zhejiang, China; ^3^Department of Coronary Care Unit, The First Affiliated Hospital, Wenzhou Medical University, Wenzhou, Zhejiang, China

## Abstract

In spite of early interventions to treat acute myocardial infarction (MI), the occurrence of adverse cardiac remodeling following heart failure due to acute MI remains a clinical challenge. Thus, there is an increasing demand for the development of novel therapeutic agents capable of inhibiting the development of pathological ventricular remodeling. RNA-seq data analysis of acute MI rat models from GEO revealed that Runx1 was the most differentially expressed MI-related gene. In this study, we demonstrated that increased Runx1 expression under pathological conditions results in decreased cardiac contractile function. We identified dihydrolycorine, an alkaloid lycorine, as a promising inhibitor of Runx1. Our results showed that treatment with this drug could prevent adverse cardiac remodeling, as indicated by the downregulation of fibrotic genes using western blotting (collagen I, TGF*β*, and p-smad3), downregulation of the apoptosis gene Bax, upregulation of the apoptosis gene Bcl-2, and improved cardiac functions, such as LVEF, LVSF, LVESD, and LVEDD. Additionally, dihydrolycorine treatment could rescue cardiomyocyte hypertrophy as demonstrated by wheat germ agglutinin staining, increased expression levels of the punctuate gap junction protein connexin 43, and decreased *α*-SMA expression, resulting in cardiomyocyte fibrosis in immunofluorescence staining. Molecular docking, binding modeling, and pull-down assays were used to identify potential dihydrolycorine-binding sites in Runx1. When Ad-sh-Runx1 was transfected into hypoxia-cardiomyocytes or injected into the hearts of MI rats, the cardioprotective effects of dihydrolycorine were abolished, and the normal electrophysiological activity of cardiomyocytes was disrupted. Taken together, the results of the present study indicate that dihydrolycorine may inhibit adverse cardiac remodeling after MI through the reduction of Runx1, suggesting that dihydrolycorine-mediated-Runx1 regulation might represent a novel therapeutic approach for adverse cardiac remodeling after MI.

## 1. Introduction

Adverse cardiac remodeling after myocardial infarction (MI) results in the development of heart failure, which is a leading cause of high mortality rates [1, 2]. Acute coronary artery blockage after MI can lead to the cardiomyocyte death and eccentric hypertrophy of surviving cardiomyocytes and initiate a reparative process involving the deposition of fibrillar collagens in infarcted and noninfarcted areas. This subsequently causes myocardial fibrosis, which induces harmful cardiac remodeling and cardiac dysfunction [3–5]. Notably, no specific medication exists to reverse adverse cardiac remodeling.

Dihydrolycorine (C_16_H_19_NO_4_), an alkaloid lycorine, is obtained by contact hydrogenation from lycoris, a derivative of the Lycoraceae family member pyrrophenthridine [6]. It has diverse biological functions, including antitumor [7–9], neuroprotective [10], antiosteoporosis [11], antiviral [12], and anti-inflammatory [13] functions, as reported by various clinical studies. Recently, for the first time, dihydrolycorine was demonstrated to exhibit cardioprotective effects [14]. However, the possible antifibrotic effects of this drug after MI should be explored, and the underlying molecular mechanisms involved in cardiac fibrosis should be elucidated.

Runt-related transcription factor-1 (Runx1), a member of the Runt-related (RUNX) family (Runx1, Runx2, and Runx3), possesses a conserved 128 aa region that mediates the function of binding to DNA *α* subunits that partner with core binding factor *β* [15] to control and guide the processes of cell differentiation, cell proliferation, and pedigree commitment in tissues [16], including the heart and vascular tissue [17, 18]. Runx1 also regulates the development and transcription during hematopoiesis [19–21]. Recently, Runx1 was demonstrated to be involved in the processes underlying adverse cardiac remodeling [22], and Runx1 activation in cardiomyocytes after MI is harmful to ventricular function [23]. However, to date, the protective role of Runx1 against adverse cardiac remodeling after MI in response to dihydrolycorine treatment remains unknown.

In this study, we explored the protective effects of dihydrolycorine in MI and elucidated the underlying mechanisms. Our data suggest that reducing the expression of Runx1 using dihydrolycorine treatment could rescue the adverse cardiac remodeling that occurs following MI, ultimately preventing the left ventricular dysfunction.

## 2. Materials and Methods

### 2.1. Ethics Statement and Surgical Ligation Model of MI

All experiments and procedures involving animals in this study were performed with humanitarian care in accordance with the Institutes of the First Affiliated Hospital of Wenzhou Medical University Health Guidelines for Laboratory Animals.

**Figure 1:**
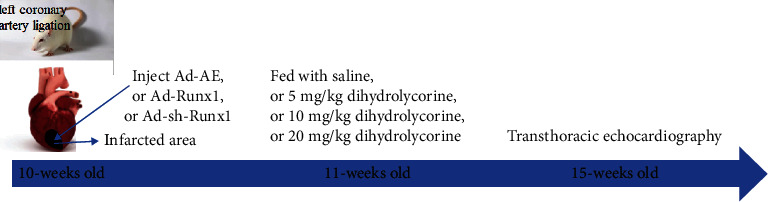
The schematic details on a temporal scale about the animal experiments.

Male SD rats (10 weeks old) were anesthetized using 2.5% isoflurane and were subjected to thoracotomy 5 mm beneath the left atrial appendage to establish a MI model. The left anterior descending coronary artery was ligated using a 9-0 suture, and the success of the surgery was confirmed by the appearance of myocardial branching. The sham control rats did not undergo left coronary artery ligation. Before closing the chest, adenovirus constructs Ad-AE, Ad-Runx1, or Ad-sh-Runx1 green fluorescent protein (GFP) were injected into the left ventricular free wall of rats in the infarcted area (10 *μ*L for each of the four sites) using a 50 *μ*L needle (705RN, USA) [24–27]. After 1-week post-MI (baseline), rats were randomly assigned to groups that received treatment with or without saline, 5 mg/kg dihydrolycorine, 10 mg/kg dihydrolycorine, and 20 mg/kg dihydrolycorine for a month (Figure 1).

### 2.2. Echocardiography

Transthoracic echocardiography was performed using a Philips iE33 system (Philips Medical, Netherlands), and left ventricular end-systolic diameter (LVESD) and left ventricular end-diastolic diameter (LVEDD) were recorded. Next, left ventricular fraction shortening (LVFS) and left ventricular ejection fraction (LVEF) were calculated using a computer algorithm after three successive cardiac cycles.

### 2.3. RNA-seq Based on Gene Expression Data Analysis

MI RNA-seq data based on the GSE114695 [28] dataset was acquired from the Gene Expression Omnibus (GEO) database (https://www.ncbi.nlm.nih.gov/geo/). Differentially expressed MI-related genes were screened using Microsoft Excel with the thresholds of ∣log2FC | >2.0 and *P* value after correction (adj.*P*.Val) < 0.05. A heat map of differentially expressed MI-related genes was constructed using GraphPad Prism 7.0.

Interacting proteins specific for the Runx1 gene were searched in the GeneCards (https://www.genecards.org/) and String databases (https://string-db.org/).

### 2.4. Molecular Docking

Molecular docking studies were performed to determine the binding mode between dihydrolycorine and human Runx1 using AutoDock Vina 1.1.2 (The Scripps Research Institute, San Diego, USA).

### 2.5. Primary Culture of Neonatal Cardiomyocytes and Cell Culture

Primary cardiomyocytes were isolated from the left ventricle of 1-day-old SD rats according to a previously described protocol [24]. Primary cardiomyocytes were cultured in a 5% CO_2_, 21% O_2_, and balanced nitrogen incubator at 37°C under normoxic conditions. Other primary cardiomyocytes were cultured in 1% O_2_, 5% CO_2_, and balanced nitrogen at 37°C (oxygen-glucose deprivation) [5, 29] to establish a MI model *in vitro* (hypoxic condition). Hypoxia cardiomyocytes were incubated with 1.0, 5.0, 10, and 15 *μ*M dihydrolycorine for 24 h. Experimental cardiomyocytes were transfected with adenovirus (Ad-Runx1, or Ad-sh-Runx1; 10 mL/mL, MOI: 100 : 1) in serum-free DMEM for 6-8 h and treated under hypoxic or normoxic conditions, with or without dihydrolycorine for an additional 24 h.

### 2.6. Immunofluorescence Staining

Experimental primary cardiomyocytes in 48-orifice-plates were fixed using 4% paraformaldehyde, permeabilized using 0.5%Triton X-100, blocked using 4% goat serum, and then incubated with primary antibodies against SM *α*-actin (*α*-SMA, ab5694), and connexin 43 (ab11370) at 4°C overnight. After incubation with DyLight 488 and 594 AffiniPure Goat IgG (H + L) for 1 h at 37°C, the cardiomyocytes were counterstained with 0.1 *μ*g/mL 4′,6-diamidino-2-phenylindole (DAPI) (P36941; Invitrogen) for 3 min and images were obtained using a Nikon Eclipse Ti-U fluorescence microscope (×400 or ×200).

### 2.7. Wheat Germ Agglutinin (WGA) Staining

WGA staining (iFluor™ 594-Wheat Germ Agglutinin Conjugate, AAT Bioquest) was performed to determine the size of cardiomyocytes and to find out whether cardiomyocytes were hypertrophic. Left ventricular tissues from infarcted areas in rat hearts were embedded in paraffin and cut into 5 *μ*m thick sections, which were then incubated with 5 *μ*g/mL WGA (L4895; Sigma-Aldrich) in the dark at room temperature and then stained with DAPI for 3 min. Images were obtained using a Nikon Eclipse Ti-U fluorescence microscope (Minato-ku, Tokyo, Japan) and analyzed using Image J software (National Institutes of Health).

### 2.8. Western Blotting

Total protein samples were obtained from left ventricular tissues from infarcted areas in rat's hearts, cardiomyocytes, and H9C2 cells (ATCC, USA). A bicinchoninic acid reagent was used according to a previously described protocol to ensure identical protein concentrations in each protein sample [30]. Protein samples were subjected to sodium dodecyl sulfate-polyacrylamide gel electrophoresis (SDS-PAGE) and then transferred onto 0.45 *μ*m polyvinylidene difluoride membranes (PVDF) (Millipore, Billerica, MA, USA). The membranes were blocked using 5% milk in TBST at room temperature for 1 h and then incubated with primary antibodies, including Runx1 (ab92336; Abcam), TGF*β* (ab215715), p-smad3 (ab52903), smad3 (ab40854), collagen I (ab34710), Bax (ab32503), Bcl-2 (ab32124), CBFB (ab133600), CEBPA (ab40761), and *β*-actin (ab8226) overnight at 4°C. PVDF membranes were incubated with horseradish peroxidase-conjugated goat anti-mouse and anti-rabbit IgG (H + L) (1 : 5000; Abbkine, Redlands, CA) on the following day and visualized using an ECL chemiluminescent kit (Sigma, United States). Quantity One 5.0 software (Bio-Rad Laboratories, Inc., Hercules, CA, USA) was used for quantification.

### 2.9. Pull-Down Assay

Binding buffer was added to the mixed lysate obtained from primary cardiomyocytes containing two proteins that predicted interaction, and the sample was then rotated for bonding determination at 4°C for 2-4 h. After centrifugation for 2 min at 4°C, the supernatant was discarded, and the cells were washed with 1 mL binding buffer 5 to 6 times. Loading buffer was added to the 20-30 *μ*L liquid that remained after the last wash. Western blotting analysis was performed.

### 2.10. Hematoxylin and Eosin (H&E) Staining

H&E staining was used to assess the degree of myocardial fibrosis and evaluate the general morphology of the myocardium. Left ventricular tissues from infarcted areas in rat hearts were embedded in paraffin and cut into 5 *μ*m thick sections, which were then incubated with hematoxylin and eosin (H&E). Images were obtained using a Nikon Eclipse Ti-U fluorescence microscope (Minato-ku, Tokyo, Japan) and analyzed using Image J software (National Institutes of Health).

### 2.11. Statistical Analyses

All experiments were repeated a minimum of three times. Data are presented as the mean ± standard deviation. Two-tailed paired or unpaired Student's *t*-tests were performed to compare two normally distributed groups with normal data distribution; one-way analysis of variance (ANOVA) or two-way ANOVA followed by Bonferroni's correction was used for multiple comparisons. If the normality of the data could not be confirmed, Mann–Whitney tests were used. All data were analyzed using SPSS version 26.0 software (SPSS Inc. USA, IL). Statistical significance was set at *P* < 0.05.

## 3. Results

### 3.1. MI-Related Genes Were Differentially Expressed, and Runx1 Was Highly Expressed after MI

**Figure 2:**
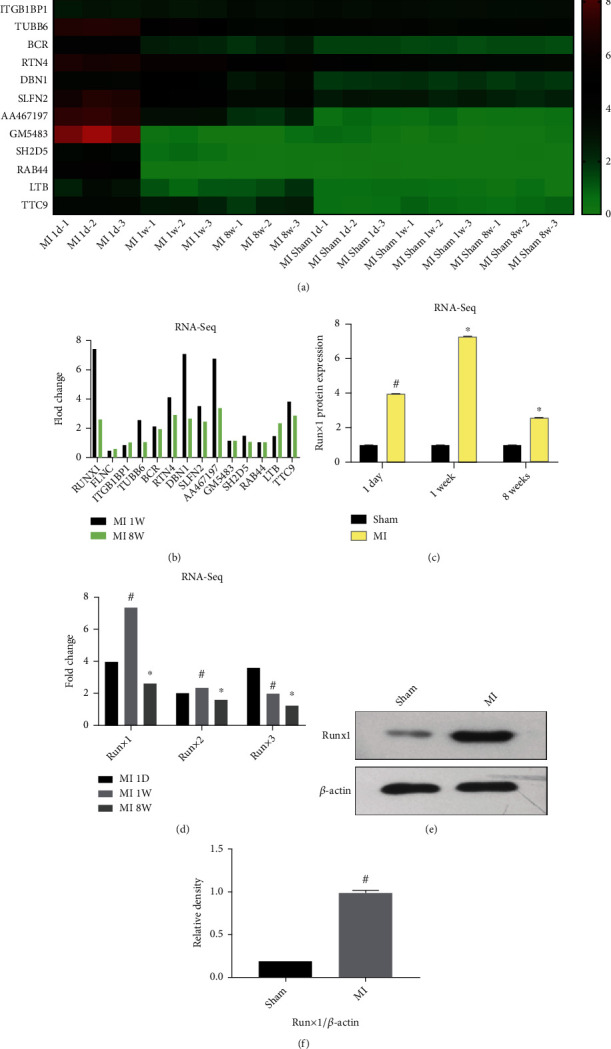
Runx1 was the most differentially expressed myocardial infarction- (MI-) related gene. (a) The heat map of the top 14 differentially expressed MI-related genes in the RNA-sequencing dataset GSE1146395. (b) Comparison of mRNA expression levels of the MI-related genes obtained from RNA-sequencing data (GSE1146395). (c) Expression of Runx1 mRNA as determined by RNA-sequencing data. (d) Comparison of the RUNX (runt-related) family mRNA levels from RNA-sequencing data. (e, f) Relative protein expression of Runx1 according to western blotting. ^#^*P* < 0.05 vs. sham and ^∗^*P* < 0.05 vs. MI; experiments were repeated more than three times.

The differentially expressed MI-related gene profiles of the infarct area were screened from the GSE1146395 dataset obtained from the GEO database based on a threshold of ∣log2FC | >2.0, and adj.*P*.Val < 0.05. The top 14 differentially expressed MI-related genes at day 1, week 1, and eight weeks after MI surgery are shown in the heat map (Figure 2(a)), with three mice in each period. After a data analysis of the RNA-sequence, we determined that Runx1 was the most differentially expressed MI-related gene (Figure 2(b)).  Figure 2(c) indicates that the highest increase in the mRNA levels of Runx1 occurred at week 1 after MI surgery compared to the levels in sham rats. In the Runt-related family (Runx1, Runx2, and Runx3), Runx1 expression was most significantly elevated (Figure 2(d)) according to the RNA-sequence analysis. We confirmed these results using western blotting and found that Runx1 was upregulated in the infarct area 1 week after MI surgery (Figures 2(e) and 2(f)).

### 3.2. Dihydrolycorine Improved Cardiac Function of MI Rats and Downregulated Runx1

**Figure 3:**
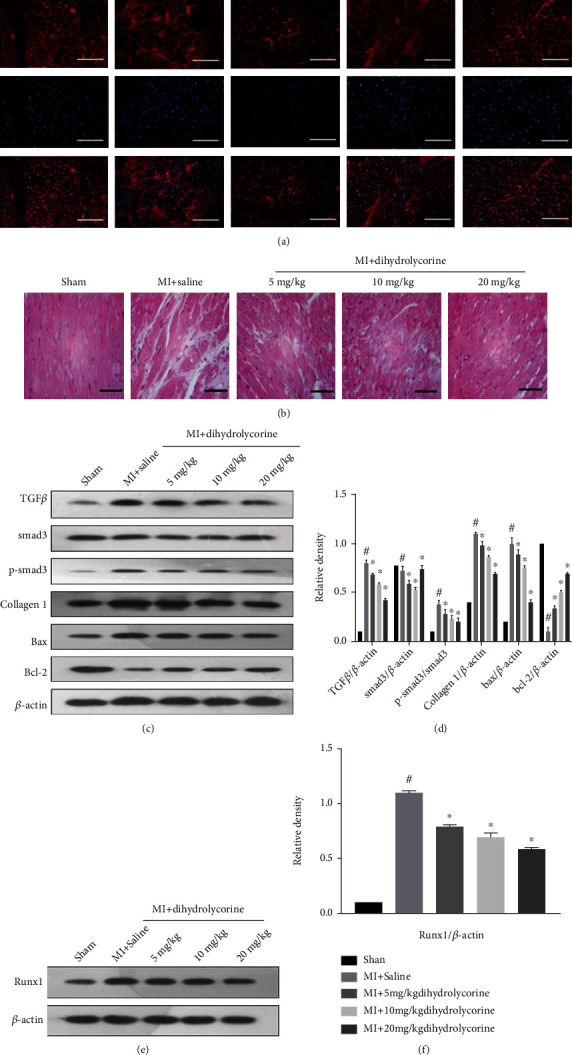
Dihydrolycorine reduced the fibrosis of the myocardium after myocardial infarction (MI). (a) Cardiac myocyte hypertrophy was determined by using wheat germ agglutinin (WGA, magnification ×100) staining of left ventricular tissue from rats. Scale bar = 100 *μ*m. (b) Myocardial fibers in myocardial tissue sections were stained with hematoxylin and eosin (H&E, magnification ×40). Scale bar = 200 *μ*m. (c, d) Relative protein expression of collagen I, TGF*β*, smad3, p-smad3, Bax, and Bcl-2 according to western blotting. (e, f) Relative protein expression with representative gel blots of Runx1 by western blot analysis. ^#^*P* < 0.05 vs. sham and ^∗^*P* < 0.05 vs. MI; experiments were repeated more than three times.

To explore the cardioprotective function of dihydrolycorine at week 1 after MI surgery and at 12 weeks after 5, 10, and 20 mg/kg dihydrolycorine treatments, we determined the cardiac function of rats using transthoracic echocardiography. As shown in Table 1, as the concentration of dihydrolycorine increased, cardiac dysfunction was further suppressed, which was indicated by increased LVEF, LVFS, and E/A ratio and decreased LVESD and LVEDD (Table 1). As expected, the left ventricular cardiomyocytes subjected to WGA staining revealed that the MI-associated enlargement of cardiomyocytes could be mitigated by treatment with a 20 mg/kg dihydrolycorine (Figure 3(a)). Further analysis revealed that dihydrolycorine can alleviate the occurrence of irregular myocardial fibers and obscure intercellular borders in the infarcted area, which showed the initiation of myocardial necrosis, as evidenced by H&E staining (Figure 3(b)). Therefore, in all the subsequent animal experiments, dihydrolycorine was administered at a concentration of 20 mg/kg. Furthermore, we observed the downregulation of fibrotic genes, including collagen I, TGF*β*, and p-smad3, in the MI rats treated with dihydrolycorine (Figures 3(c) and 3(d)), downregulation of the apoptosis gene Bax, and upregulation of the apoptosis gene Bcl-2. Most importantly, upregulation of Runx1 after MI surgery was inhibited by dihydrolycorine treatment (Figures 3(e) and 3(f)). These results confirmed that *in vitro* dihydrolycorine treatment could prevent MI-induced myocardial fibrosis and cardiomyocyte hypertrophy and may thus mediate Runx1 to exert a cardioprotective effect.

### 3.3. Dihydrolycorine May Directly Inhibit Runx1 and Enhance Its Activity

**Figure 4:**
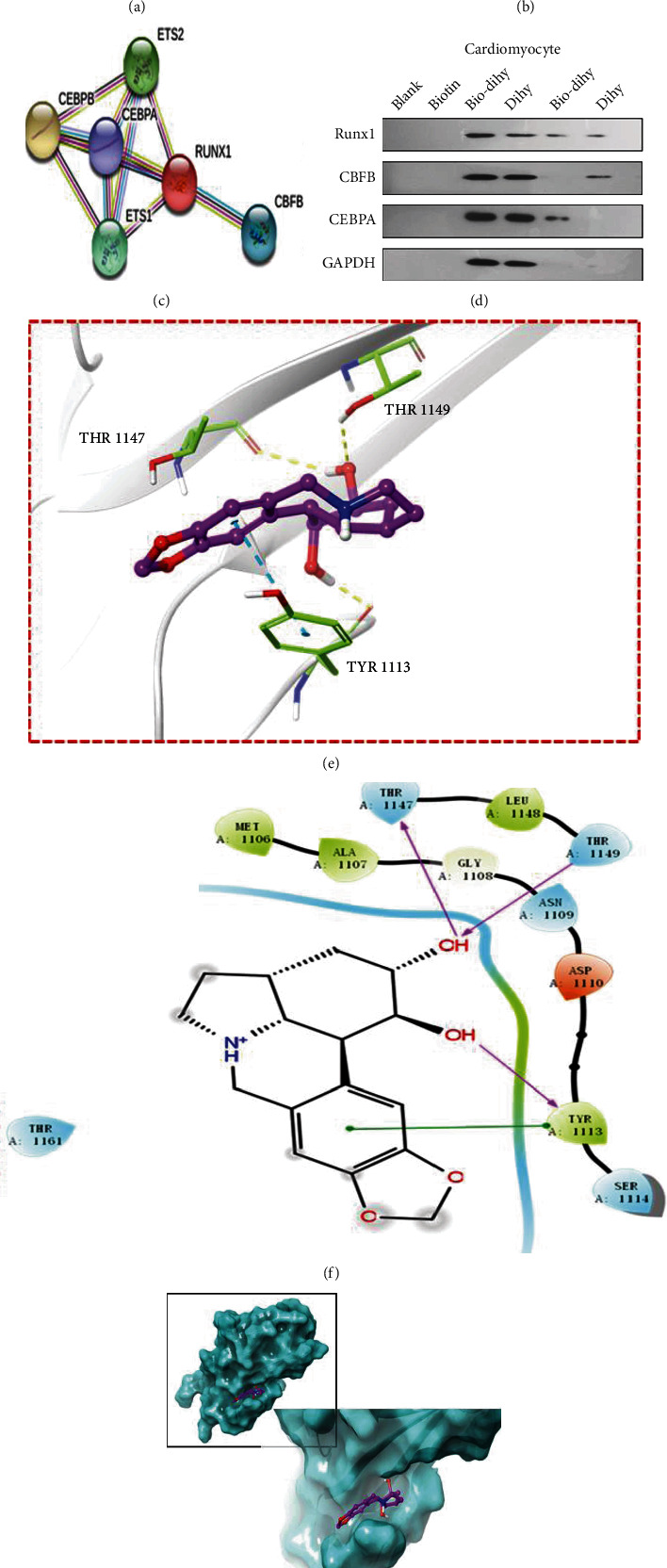
Identification of dihydrolycorine binding proteins. (a) Protein expression with representative gel blots of Runx1 according to western blotting. Lysates prepared from (a) rats' ventricle tissues and (b) H9C2 cells were added to the streptavidin-agarose beads with Bio-Dihy that were previously added to the streptavidin-agarose beads with prior incubation. (c) The upstream signaling proteins of Runx1 according to GeneCards and the STRING Interaction Network. (d) Pull-down assay was used to assess dihydrolycorine binding to Runx1, CBFB, and CEBPA. Lysates were obtained from primary cardiomyocytes. (e–g) Representative images were obtained by molecular docking between dihydrolycorine and Runx1.

To determine whether dihydrolycorine binds to Runx1 in cardiomyocytes, we performed biotinylated protein interaction pull-down assays. Biodihydrolycorine (Dihy) was incubated with streptavidin-agarose beads, and lysates from the left ventricle tissues of rats and H9C2 cells were added. Biodihydrolycorine binds to Runx1 protein in lysates from both rats' left ventricle tissue and H9C2 cells (Figures 4(a) and 4(b)). Furthermore, when we explored upstream signaling proteins of Runx1 (CBFB and CEBPA), which were screened by GeneCards and the STRING Interaction Network (Figure 4(c)), we found that these proteins did not interact with dihydrolycorine according to the results of the pull-down assay (Figure 4(d)). These results demonstrate that dihydrolycorine directly binds to Runx1 rather than to its upstream mediators.

To explore the interaction between Runx1 and dihydrolycorine, we performed molecular docking using AutoDock Vina 1.1.2. and determined the crystal structure of Runx1 (PDB ID:1LJM) using a simulation study.  Figure 4(e) shows three binding sites on Runx1, including THR 1147, THR 1149, and TYR 1113, which form three hydrogen bond interactions and one Pi-Pi interaction with TYR 1113, with an affinity of -6.1 kcal/mol. The chemical structure of the binding site between dihydrolycorine and Runx1 is shown in Figure 4(f).  Figure 4(g) shows the crystal structure of the binding site between dihydrolycorine and Runx1 in the three-dimensional diagram.

### 3.4. Dihydrolycorine Exerted Positive Effects on Hypoxia-Treated Cardiomyocytes

**Figure 5:**
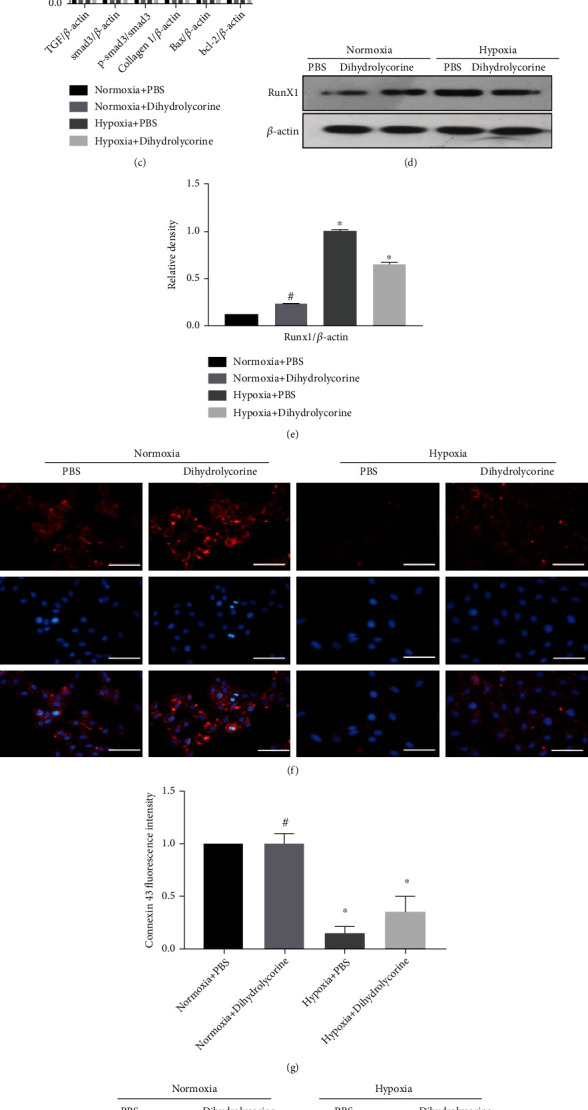
Dihydrolycorine rescued fibrosis and apoptosis in hypoxia-treated cardiomyocytes and induced decreased Runx1 expression. (a) Hypoxia-treated cardiomyocytes were treated with 1.0, 5.0, or 10 *μ*M dihydrolycorine for 24 h, followed by DNA quantification to measure cell number/cell proliferation. (b, c) Relative protein expression of collagen I, TGF*β*, smad3, p-smad3, Bax, and Bcl-2 according to western blotting. (d, e) Relative protein expression of Runx1 according to western blotting. (f) Immunofluorescence staining of connexin 43 (red, magnification ×400). Scale bar = 50 *μ*m. (g) Bar graphs of fluorescence intensity of connexin 43. (h) Immunofluorescence staining of *α*-SMA (green, magnification ×400). Scale bar = 50 *μ*m. ^#^*P* < 0.05 vs. normoxia and ^∗^*P* < 0.05 vs. hypoxia; experiments were repeated more than three times.

We confirmed that dihydrolycorine exhibited cardioprotective effects *in vitro*. First, we found that the number of hypoxia-treated cardiomyocytes was significantly higher following the culture with dihydrolycorine at concentrations of 1.0, 5.0, 10, and 15 *μ*M for 24 h than that observed in the control group; however, these numbers began decreasing upon at treatment with 15 *μ*M dihydrolycorine (Figure 5(a)). For all subsequent cell experiments, dihydrolycorine was added at a concentration of 10 *μ*M. Second, we observed that the expression levels of fibrotic genes and apoptosis genes, including collagen I, TGF*β*, p-smad3, and Bax, were decreased in the dihydrolycorine-treated hypoxia-cardiomyocytes (Figures 5(b) and 5(c)). Third, Runx1 was also downregulated in dihydrolycorine-treated hypoxia-treated cardiomyocytes (Figures 5(d) and 5(e)). Fourth, we identified that the cardiac functional protein, punctuate gap junction protein connexin 43 was rescued by dihydrolycorine treatment in hypoxia-cardiomyocytes in proximity to the plasma membrane according to immunofluorescent staining results (Figures 5(f) and 5(g)). Finally, we investigated the overexpression of *α*-SMA in hypoxia-cardiomyocytes that resulted in cardiomyocyte fibrosis as assessed by immunofluorescence staining and found that the levels of this protein were reduced after dihydrolycorine treatment (Figure 5(h)). Taken together, these results suggest that dihydrolycorine prevents hypoxia-induced cardiomyocyte fibrosis and regulates the expression of Runx1.

### 3.5. Dihydrolycorine Suppressed Myocardial Fibrosis after MI by Reducing Runx1 Levels *In Vivo* and *In Vitro*

**Figure 6:**
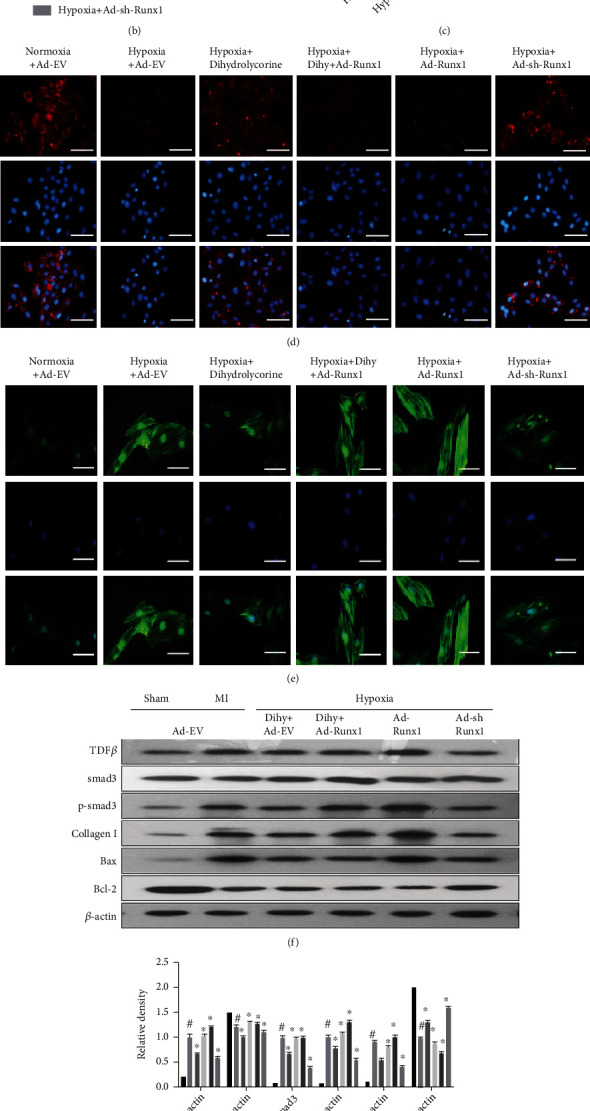
(a–e) Dihydrolycorine mitigated adverse cardiac remodeling after myocardial infarction (MI) reducing Runx1 *in vivo* and *in vitro*. Ad-EV, Ad-Runx1, and Ad-sh-Runx1 (10 mL/mL, MOI: 100 : 1) were transfected into experimental cardiomyocytes for 6-8 hours, and these cells were treated with or without dihydrolycorine (15 *μ*M) under hypoxia conditions. MI rats (*n* = 8) and sham rats (*n* = 8) were injected with Ad-EV, Ad-Runx1, and Ad-sh-Runx1 (10 mL at each of four sites, 1.2 × 10^10^ PFU/mL) into the left ventricle free wall and then treated with or without dihydrolycorine (20 mg/kg). (a, b) Relative protein expression of collagen I, TGF*β*, smad3, p-smad3, Bax, and Bcl-2 according to western blotting. (c) Immunofluorescence staining of connexin 43 (red, magnification ×400). Scale bar = 50 *μ*m. (d) Bar graphs of fluorescence intensity of connexin 43. (e) Immunofluorescence staining of *α*-SMA (green, magnification ×400). Scale bar = 50 *μ*m. (f–i) (f, g) Relative protein expression of collagen I, TGF*β*, smad3, p-smad3, Bax, and Bcl-2 according to western blotting. (h) Cardiac myocyte hypertrophy was evaluated using WGA (magnification ×100) staining of left ventricular tissue from rats. Scale bar = 100 *μ*m. (i) Myocardial fibers in myocardial tissue sections were stained with hematoxylin and eosin (H&E, magnification ×40). Scale bar = 200 *μ*m. ^#^*P* < 0.05 vs. normoxia and ^∗^*P* < 0.05 vs. hypoxia; or ^#^*P* < 0.05 vs. sham and ^∗^*P* < 0.05 vs. MI; experiments were repeated more than three times.

We next investigated the specific mechanisms underlying the positive effect of dihydrolycorine in MI rats and hypoxia-treated cardiomyocytes, while confirming whether Runx1 plays an indispensable role in this process. As shown in Figures 6(a) and 6(b) (*P* < 0.05), there was a significant downregulation of fibrotic genes and apoptosis genes in the Ad-sh-Runx1+hypoxia-treated cardiomyocytes and dihydrolycorine+hypoxia-treated cardiomyocytes. Administration of Ad-Runx1 significantly abolished the cardioprotective function of dihydrolycorine, as demonstrated by the increased expression of the cardiomyocyte fibrosis gene *α*-SMA and decreased expression of punctuate gap junction protein connexin 43 in immunofluorescence staining (Figures 6(c)–6(e)). Moreover, Ad-Runx1, which was injected into the left ventricle of MI rats, could offset the cardioprotective effect of dihydrolycorine, as characterized by increased in LVEDD and LVESD and decreased LVEF and LVFS compared to the MI+dihydrolycorine+Ad-EV group (Table 2). As expected, the stimulation of Runx1 in MI rats failed to rescue myocardial fibrosis as measured by the expression of fibrotic and apoptotic genes (Figures 6(f) and 6(g)) and was unable to mitigate cardiomyocyte hypertrophy even after dihydrolycorine treatment (Figure 6(h)). H&E staining confirmed that in the MI+Ad-Runx1 group and the MI+dihydrolycorine+Ad-Runx1 group, irregular myocardial fibers and obscure intercellular borders were trapped. In comparison to the MI+dihydrolycorine group or the MI+Ad-sh-Runx1 group, the cardiac fibers were rearranged. Taken together, these results confirm Runx1 directly influences the cardioprotective effect of dihydrolycorine in hypoxia-treated cardiomyocytes and MI rats.

## 4. Discussion

MI subsequently triggers necrotic myocardium and myocardial fibrosis due to hypoxia caused by sudden blood flow occlusion of the coronary artery [31]. This, in turn, induces excessive and progressive remodeling of the cardiac infarct tissue and remote myocardium, ultimately leading to ventricular dysfunction and the clinical syndrome of heart failure [32, 33]. Depending on the application of early percutaneous coronary intervention, premature mortality can dramatically decrease [34]. Unfortunately, the incidence of heart failure and rehospitalization after MI has increased in recent decades because this surgery only reduces symptoms [35, 36]. However, the underlying mechanisms of adverse cardiac remodeling after MI remain unclear. Furthermore, no specific therapy is available to cure or alleviate adverse cardiac remodeling after MI. In this study, we identified the cardioprotective effect of dihydrolycorine against adverse cardiac remodeling after MI and found that Runx1 is a critical regulator of myocardial fibrosis in the occurrence and development of adverse cardiac remodeling.

Recently, several studies have reported that Runx1 may be a promising therapeutic target for cardiovascular diseases [23, 37, 38]. Runx1 directly targets phosphatidylcholine transfer proteins in patients with cardiovascular diseases [39]. Runx1 induces endogenous heart repair by controlling the regenerative response of zebrafish cardiac cells [40]. Initially, we determined that Runx1 was significantly upregulated and was the most differentially expressed MI-related gene after MI surgery based on MI RNA-seq data obtained from the GSE1146395 dataset from GEO (Figures 2(a)–2(d)). Consistent with this, western blotting revealed that Runx1 expression increased after MI (Figures 2(e) and 2(f)). However, the ability of Runx1 to function as a regulator of adverse cardiac remodeling after MI remains unknown.

It has been previously reported that dihydrolycorine exerts a positive effect on adverse cardiac remodeling after MI, and the Natural Compound Library Screening identified dihydrolycorine as a candidate drug against cardiac fibrosis, as reported by Schimmel et al. in 2020 [14]; however, its ability to attenuate cardiac fibrosis after MI remains unknown. Surprisingly, we found that dihydrolycorine could rescue cardiac function after MI, as observed by increased LVEF and LVFS and decreased LVEDD and LVESD (Table 1). Moreover, fibrotic genes such as collagen I, TGF*β*, and p-smad3 and the apoptosis gene Bax were downregulated in the left ventricles of dihydrolycorine-treated MI rats (Figures 3(c) and 3(d)), thus preventing cardiomyocytes from becoming hypertrophic (Figure 3(a)) and irregular myocardial fibers and obscure intercellular borders in infarct areas (Figure 3(b)). Interestingly, there is decreased expression of Runx1 in the left ventricle tissues of dihydrolycorine-treated MI rats (Figures 3(e) and 3(f)).

Subsequently, we also demonstrated that Runx1 is the binding protein for dihydrolycorine through biotinylated protein interaction pull-down assays (Figures 4(a)–4(d)) and molecular docking analyses (Figures 4(e)–4(g)). Moreover, we observed that long-term hypoxia led to the upregulation of fibrotic and apoptotic gene expression in cardiomyocytes, which could be attenuated by treatment with dihydrolycorine (Figures 5(b) and 5(c)). Disruption of *α*-SMA and the augmentation of connexin 43, as shown by immunofluorescence staining results, in dihydrolycorine-treated hypoxia-cardiomyocytes indicated a reduction in myocardial fibrosis when compared with the hypoxia-cardiomyocyte group. As expected, Runx1 levels were lower in dihydrolycorine-treated hypoxia-cardiomyocytes than in the control group (Figures 5(d) and 5(e)). Finally, we found that stimulation of Runx1 in dihydrolycorine-treated hypoxia-cardiomyocytes abolished the cardioprotective effect of dihydrolycorine, as demonstrated by the increased expression of fibrotic and apoptotic genes (Figures 6(a) and 6(b)), the augmentation of *α*-SMA, and reduction of connexin 43 (Figures 6(c)–6(e)) compared with the hypoxia+dihydrolycorine+Ad-EV group. Concurrently, Runx1 stimulation counteracted the cardioprotective effects of dihydrolycorine, as demonstrated by a decrease in LVEF and LVFS, an increase in LVEDD and LVESD, and aggravation of cardiomyocyte hypertrophy (Figures 6(g) and 6(h)) and irregular myocardial fibers and obscure intercellular borders in infarct areas (Figure 6(i)) compared with the MI+dihydrolycorine+Ad-EV group (Table 2).

## 5. Conclusions

**Figure 7:**
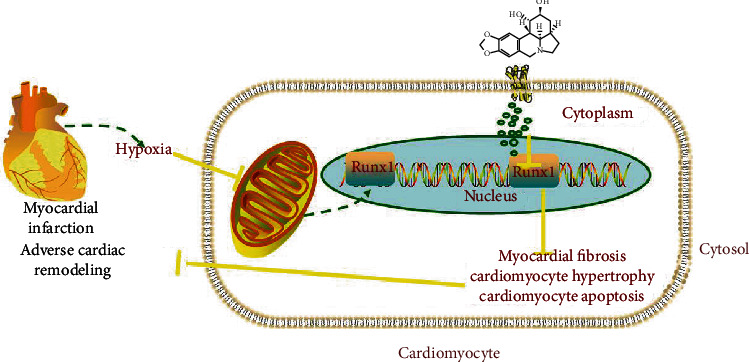
Schematic illustration indicating the cardioprotective effects of dihydrolycorine on myocardial infarction (MI) induced adverse cardiac remodeling.

In summary, in the present study, we found that dihydrolycorine could alleviate myocardial fibrosis progression, leading to adverse cardiac remodeling after MI. This was associated with decreased expression of Runx1 (Figure 7). Thus, this study demonstrated that dihydrolycorine might be a promising agent for cardiac antifibrotic therapy after MI.

## Figures and Tables

**Table 1:** Dihydrolycorine improved the cardiac function in the hearts of MI rats.

Group (*n* = 8)	Sham	MI+saline	MI+dihydrolycorine		
			5 mg/kg	10 mg/kg	20 mg/kg
LVEF (%)	79.39 ± 1.21	50.32 ± 3.53	53.13 ± 2.03^∗^^#^	57.36 ± 1.29^∗^^#^	64.23 ± 3.62^∗^^#^
LVFS%	39.1 ± 1.3	21.2 ± 3.5	24.3 ± 4.1^∗^	28.1 ± 2.6^∗^^#^	31.3 ± 2.4^∗^^#^
LVESD (mm)	2.4 ± 0.31	3.6 ± 0.42	3.4 ± 0.23^∗^^#^	3.2 ± 0.34^∗^^#^	2.9 ± 0.23^∗^^#^
LVEDD (mm)	3.7 ± 0.3	4.9 ± 0.6	4.6 ± 0.3^∗^^#^	4.3 ± 0.4^#^	4.0 ± 0.2^∗^^#^
E/A ratio	2.13 ± 0.56	1.48 ± 0.36	1.62 ± 0.41^∗^^#^	1.73 ± 0.32^∗^^#^	1.83 ± 0.36^∗^^#^
HR (bmp)	520 ± 8	480 ± 5	487 ± 2^∗^	492 ± 4^∗^^#^	501 ± 7^∗^^#^

Values are mean ± SD, *P* < 0.05. The data are presented as means and SD. ^∗^*P* < 0.05 versus sham; ^#^*P* < 0.05 versus MI + saline. LVEDD: left ventricular end-diastolic dimension; LVESD: left ventricular end-systolic dimension; LVEF: left ventricular ejection fraction; LVFS: left ventricular systolic function; HR: heart rate.

**Table 2:** Dihydrolycorine fails to exert protective effects in MI rats injected with Ad-Runx1.

Group (*n* = 8)	Sham+Ad-EV	MI+Ad-EV	MI+Dihy+Ad-EV	MI+Dihy+Ad-Runx1	MI+Ad-Runx1	MI+Ad-sh Runx1
LVEF (%)	79.26 ± 2.13	50.36 ± 1.45	64.16 ± 2.64^∗^^#^	51.32 ± 3.63^∗^^#^	48.41 ± 2.61^∗^^#^	65.37 ± 3.14^∗^^#^
LVFS%	39.3 ± 1.4	21.5 ± 3.2	31.6 ± 2.3^∗^^#^	21.9 ± 4.1^∗^	20.8 ± 3.8^∗^^#^	31.9 ± 2.6^∗^^#^
LVESD (mm)	2.4 ± 0.26	3.6 ± 0.41	2.9 ± 0.35^∗^^#^	3.2 ± 0.12^∗^^#^	3.8 ± 0.32^∗^^#^	2.8 ± 0.38^∗^^#^
LVEDD (mm)	3.7 ± 0.4	4.9 ± 0.4	4.0 ± 0.4^∗^^#^	4.8 ± 0.2^#^	5.1 ± 0.6^∗^^#^	3.9 ± 0.3^∗^^#^
E/A ratio	2.14 ± 0.36	1.48 ± 0.45	1.83 ± 0.48^#^	1.46 ± 0.61^∗^^#^	1.42 ± 0.21^∗^^#^	1.86 ± 0.36^∗^
HR (bmp)	520 ± 8	480 ± 5	502 ± 5^∗^^#^	478 ± 4^∗^^#^	469 ± 9^∗^	506 ± 7^∗^^#^

Values are mean ± SD, *P* < 0.05. The data are presented as means and SD. ^∗^*P* < 0.05 versus sham+Ad-EV; ^#^*P* < 0.05 versus MI+Ad-EV. LVEDD: left ventricular end-diastolic dimension; LVESD: left ventricular end-systolic dimension; LVEF: left ventricular ejection fraction; LVFS: left ventricular systolic function; HR: heart rate.

## Data Availability

MI RNA-seq data based on the GSE1146395 [27] dataset was acquired from the Gene Expression Omnibus (GEO) database (https://www.ncbi.nlm.nih.gov/geo/). Other data are available upon request (contact the corresponding author).
